# Bioprinted core-shell living material platform for spatially controlled encapsulation of *Bacillus subtilis* and sustained metabolite exchange

**DOI:** 10.1016/j.bioactmat.2026.08.025

**Published:** 2026-08-31

**Authors:** Lin Huang, Kathleen L. Furtado, William Brakewood, Maxwell Neal, Yazhi Sun, Jasmine Le, Qi Xie, Jacob Hizon, Shivam Singhal, Mariana Salas Garcia, Joshua Tran, Karsten Zengler, Michael Betenbaugh, Jack A. Gilber, Shaochen Chen

**Affiliations:** a Department of Chemical and Nano Engineering, University of California San Diego, La Jolla, USA; b Department of Pediatrics, University of California San Diego, La Jolla, CA, USA; c Scripps Institution of Oceanography, University of California San Diego, La Jolla, CA, USA; d Department of Chemical and Biomolecular Engineering, Johns Hopkins University, Baltimore, MD, USA; e Department of Bioengineering, University of California San Diego, La Jolla, USA; f Department of Chemistry, Johns Hopkins University, Baltimore, MD, USA; g Center for Microbiome Innovation, University of California San Diego, La Jolla, CA, USA

**Keywords:** 3D bioprinting, Engineered living materials, Biocontrol, Hydrogels, Antimicrobial resistance, MRSA, *Bacillus subtilis*, Pathogens

## Abstract

Antimicrobial resistance (AMR) represents an escalating global health crisis, demanding alternative strategies to reduce resistant pathogen burden across environments. Microbe-based biocontrol is promising, yet effectively deploying it in practical settings remains challenging. In this study, we present a 3D bioprinted core-shell construct featuring a polyethylene glycol diacrylate (PEGDA) shell with tunable nanoscale porosity, encapsulating germinable spores of the biocontrol agent *Bacillus subtilis* TH035. This configuration supports long-term spore viability while providing protection from common environmental stressors including UV-C irradiation, ethanol exposure, and desiccation over 4 weeks. The nanoporous PEGDA shell enables effective bacterial confinement while facilitating sufficient metabolite exchange for *B. subtilis* germination and growth, as well as suppression of methicillin-resistant *Staphylococcus aureus* (MRSA) growth by approximately one order of magnitude. This approach demonstrates the feasibility of embedding *B. subtilis* spores within engineered scaffolds for extended competitive functionality. The versatility and scalability of digital light processing (DLP) based bioprinting offers significant potential for tailored designs and high-throughput manufacturing. This proof-of-concept platform may find future applications in areas such as biomedical packaging, environmental sanitation, and built environment surface coatings, particularly in settings where intermittent moisture or nutrient availability can support spore germination and biocontrol activity.

## Highlights

• Bioprinted living materials enable sustained pathogen biocontrol.
• Core–shell design achieves biocontainment with functional metabolite exchange.
• Encapsulated *B. subtilis* suppresses MRSA without compromising biosafety.
• Resilient platform maintains biocontrol function under repeated stress.
• Scalable DLP fabrication offers potential for diverse environmental applications.

## Graphical abstract

**Figure:**
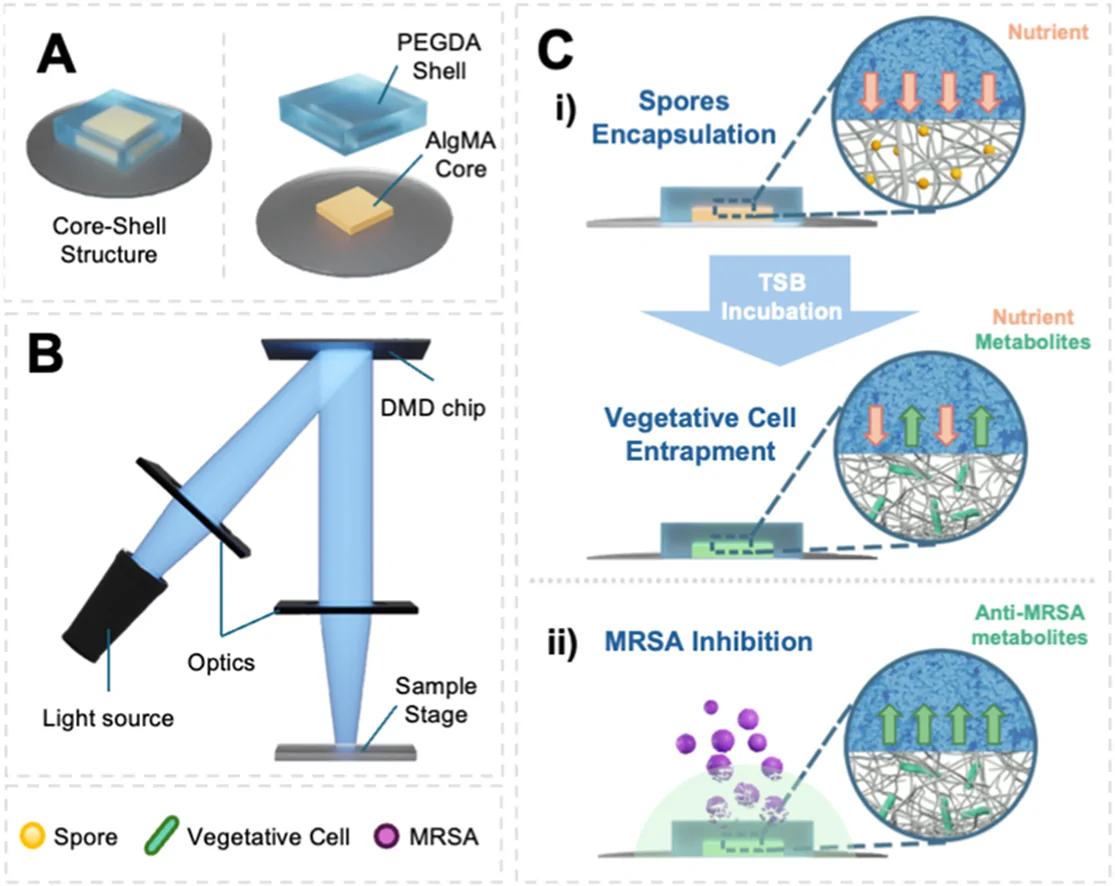

## 1 Introduction

The rise of antimicrobial resistance (AMR) in human pathogens poses a critical and growing threat to global public health []. Among the most concerning AMR pathogens is methicillin-resistant *Staphylococcus aureus* (MRSA), as efforts to reduce MRSA infection rates in U.S. hospitals have plateaued. MRSA is frequently transmitted to people via contact with contaminated surfaces [1,2]. Current approaches to reduce pathogen burden on surfaces rely on frequent and prolonged use of chemical disinfectants, which may accelerate resistance development in built environment bacteria, necessitating the development and validation of alternative pathogen biocontrol methods [3,4].

Biological approaches to pathogen control have gained attention as alternatives to conventional antimicrobial cleaning products [[5], [6], [7]]. Previous work suggests that species of *Bacillus* can actively control potentially pathogenic *Pseudomonas* on built environment surfaces [8], and cleaning products formulated with spores derived from multiple *Bacillus species* have already been commercialized for active biocontrol [9]. One of these commercial formulations, containing *Bacillus subtilis*, *Bacillus pumilus* and *Bacillus megaterium* spores, demonstrated significant efficacy in reducing MRSA burdens by 50% to 89% on hard surfaces compared to standard disinfecting regimes [10]. However, these applications rely upon frequent reapplication of *Bacillus* spores for sustained biocontrol, which limits their long-term practicality. Encapsulating viable spores within engineered material scaffolds could potentially address this limitation by providing a platform for maintaining viable microbial populations over extended periods, reducing the need for repeated manual applications. Beyond prolonged viability, encapsulation within a defined material architecture offers additional potential advantages over direct spore application: physical containment of germinated bacteria to mitigate the risk of unintended environmental release, a concern that has been raised for both engineered and unedited biocontrol strains, and structural protection of the embedded microbial population against common environmental stressors [11,12].

3D-printing has revolutionized manufacturing across sectors such as biotechnology, medicine, electronics, and energy by offering higher design flexibility for greater customization, faster time-to-product potential with reduced costs, less need for capital investment, and reduced dependence on global supply chains [13,14]. Recently, bioprinting technologies have been employed to fabricate living materials embedded with microorganisms, enabling programmable functionality in applications ranging from wound healing and bioelectronic infection control to bioplastics and biofuels [[15], [16], [17], [18], [19]]. Diverse and frequently genetically engineered microorganisms such as *Escherichia coli*, *Synechococcus elongatus*, and *Saccharomyces cerevisiae* can be genetically engineered and integrated into 3D printing pipelines to support the creation of biofriendly and programmable living materials capable of performing multiple functions in dynamic environments.

Leveraging the inherent resilience of bacterial spores to heat, desiccation, and ultraviolet radiation, several groups have incorporated *Bacillus* spores into engineered material platforms, including silica biocomposites with self-assembling protein scaffolds and bacterial cellulose matrices with dormant spores that germinate on demand [20,21]. In the context of additive manufacturing, Voigt et al. demonstrated that *Bacillus* spores can be directly incorporated into materials via extrusion-based bioprinting. In this approach, spores from genetically engineered *B. subtilis* can survive passage through high-temperature nozzles, and subsequently germinate and synthesize target compounds in response to programmed environmental cues [22]. However, this system employs an agarose-based hydrogel, which is unsuitable for high-contact areas, high-traffic public spaces, and settings with elevated biohazard concerns. In another notable study, Connell et al. introduced a method to fabricate microscopic 3D bacterial chambers using top-down multiphoton lithography and bovine serum albumin as the base material. This technique enabled the encapsulation of distinct microbial populations in core–shell architectures, facilitating controlled interactions within bacterial communities [23]. Tang et al. established a robust, multilayered “tough shell” system for sensing and computation, but the manual fabrication process and inherent cytotoxicity of the chemical crosslinkers limit both production scalability and immediate post-print viability [24].

Recent risk assessments by the U.S. Environmental Protection Agency and studies in applied microbiology have emphasized that the release of genetically engineered strains necessitates robust biocontainment strategies to prevent unintended proliferation, mitigate ecological impacts, and address regulatory and public acceptance barriers [11,12]. In addition, even unedited strains of *Bacillus* or other biocontrol agents could still harbor or transfer antimicrobial resistance genes to pathogens. To address these limitations, it is necessary to develop advanced material systems that ensure biocontainment despite frequent contact and exposure to common environmental stressors, while simultaneously allowing for long-term biological activity.

**Fig. 1:**
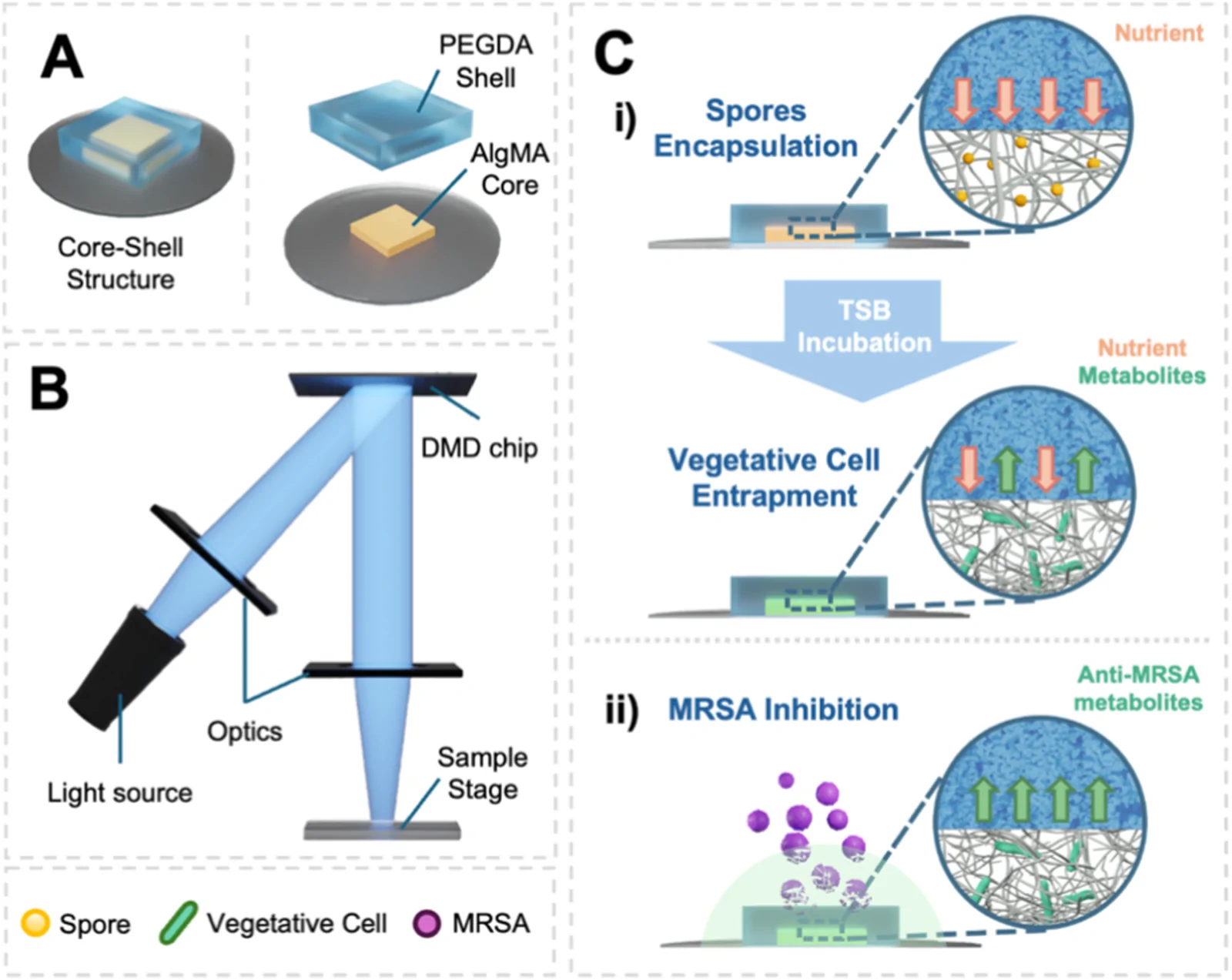
Overview of the DLP-based bioprinting strategy for constructing a core–shell living material. (A) Core–shell architecture enables selective diffusion of nutrients and metabolites through a PEGDA shell while physically isolating the AlgMA biological core. (B) Schematic of the DLP bioprinter setup consisting of a 385 nm LED light source, optics, a digital micromirror array device (DMD), and a 3D stage with computer control. (C) Illustration of functional components in the bioactive material: (i) *B. subtilis* spores embedded in the AlgMA core germinate upon incubation in tryptic soy broth (TSB), forming vegetative cells that remain spatially confined within the shell. Nutrients diffuse inward while bacterial metabolites and antimicrobial compounds diffuse outward. (ii) MRSA Inhibition. Metabolite exchange, such as the release of antimicrobial metabolites and sequestration of nutrients, suppress surrounding MRSA cells and highlight the potential for sustained and passive pathogen control in surface environments with these constructs.

In this study, we designed a core–shell structure fabricated by 3D bioprinting that moves beyond simple spore inclusion, where spores are mixed into a bulk material without physical barriers, toward true spore encapsulation with biocontainment, in which a structurally defined shell physically confines germinated bacteria while selectively permitting metabolite exchange with the surrounding environment. *B. subtilis* was selected as the microbial agent as it is genetically tractable and widely used in probiotic applications. It has the capacity to produce a broad spectrum of antimicrobial compounds with multiple modes of action and demonstrated activity against pathogens like MRSA [25,26], and it is able to outcompete pathogens by occupying surfaces (e.g., via biofilm formation) and consuming nutrients. In our design, *B. subtilis* spores are encapsulated in an alginate methacrylate (AlgMA) hydrogel core material and polymerized through digital light processing (DLP)-based bioprinting to ensure structural integrity and long-term biological viability. For the shell, we used polyethylene glycol diacrylate (PEGDA) mixed with methanol as a porogen to generate pores with tunable nanoscale dimensions (Fig. 1). These pores enable the diffusion of small metabolites, such as nutrients and secreted antimicrobial compounds, while effectively containing the vegetative cells that emerge following germination and preventing their release into the environment. This approach illustrates the potential for integrating living, functional materials into engineered platforms, opening future directions for sustained antimicrobial strategies adaptable to diverse environmental and health-related challenges.

## 2 Results

### 2.1 Optimization of a hydrogel core with encapsulated spores

**Fig. 2:**
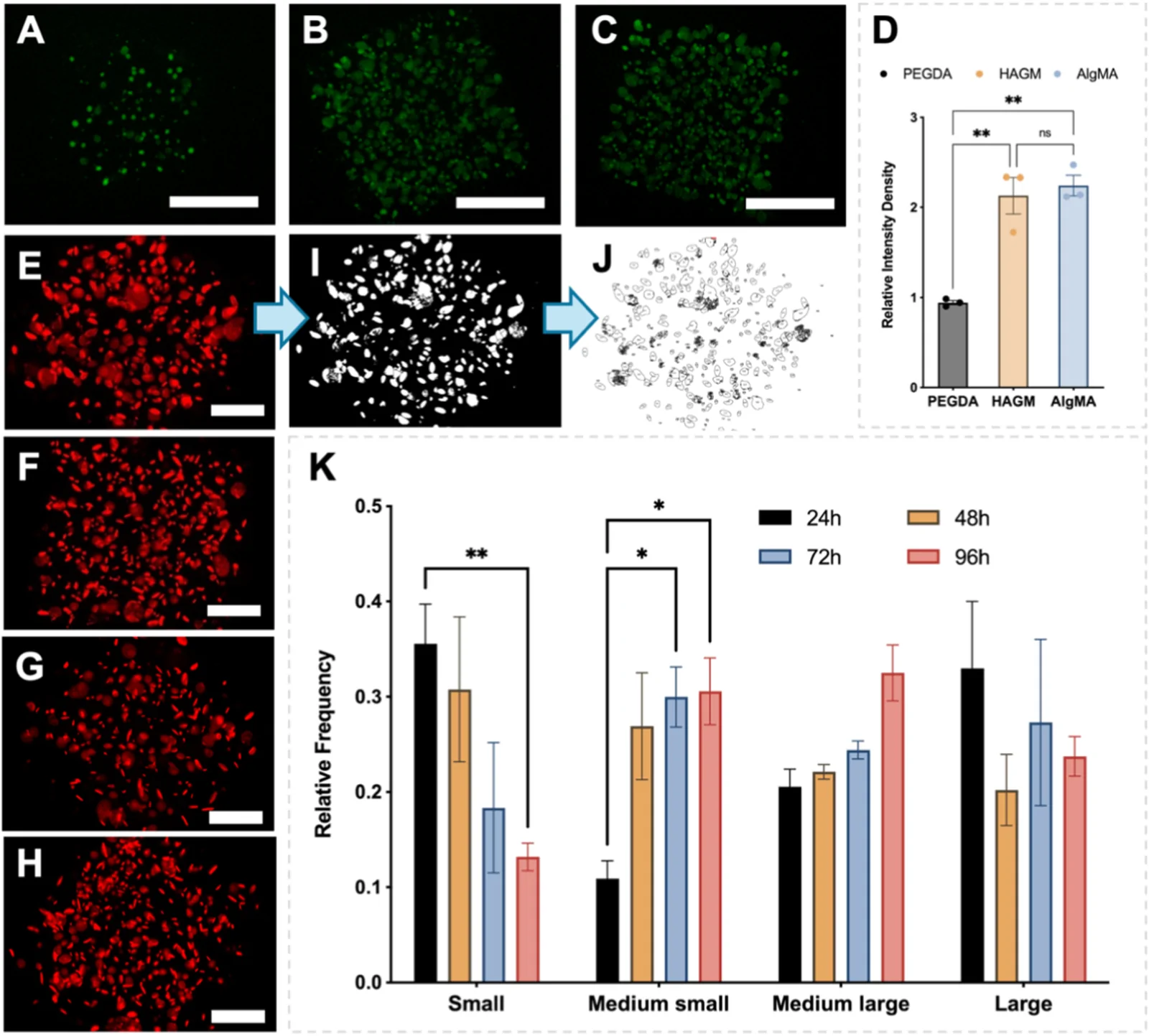
(A–C) Representative GFP fluorescence images showing germinated *B. subtilis* spores encapsulated within PEGDA 700, HAGM, and AlgMA hydrogels, respectively. Scale bar: 500 μm. (D) Quantification of relative GFP fluorescence intensity for spores encapsulated in PEGDA 700, HAGM, and AlgMA, indicating differences in germination efficiency and bacterial viability (**P < 0.01 mean ± s.e.m, n = 3). (E–H) SYTOX™ Orange staining of bacteria germinated within AlgMA hydrogels at 24, 48, 72, and 96 h post-inoculation. Scale bar: 250 μm. (I&J) Representative image analysis pipeline for quantifying colony size at the 24-h time point in AlgMA hydrogel. (K) Relative frequency distribution of bacterial colony sizes grouped by quartiles (quartiles defined by dividing the aggregate size distribution into four equal-frequency bins at each condition; see Section 4 for details), showing changes in colony size distribution over time (**P < 0.01, *P < 0.05; mean ± s.e.m, n = 3). Only statistically significant differences are marked; unmarked comparisons were not significant.

The spore-encapsulating material selected for this study was chosen based on its biocompatibility and printability, with the aim of ensuring high bacterial viability post-germination and adequate printing resolution. We initially evaluated three commonly used DLP bioprinting materials for their ability to support spore germination and maintain bacterial viability [13,27]: the synthetic polymer PEGDA and two chemically modified natural polymers, AlgMA and hyaluronic acid glycidyl methacrylate (HAGM). Each of these materials was embedded with spores from *B. subtilis* TH035, a soil isolate, with IPTG inducible GFP expression (*B. subtilis* TH035 (pSC01-GFP)). Both HAGM and AlgMA supported significantly greater bacterial germination and outgrowth from the material, as indicated by higher GFP fluorescence intensity, compared to PEGDA (Fig. 2A–D). While HAGM and AlgMA demonstrated similar capabilities in promoting bacterial viability, AlgMA offered better printing quality with cleaner edges. Based on this combination of biological and fabrication performance, AlgMA was selected as the encapsulation material for subsequent experiments.

We further optimized light exposure parameters for core fabrication to maximize bacterial viability post-germination. Our results indicate that increasing the light intensity from the minimum printable threshold did not result in significant differences in either the mechanical properties of the printed constructs (Fig. S1A) or the viability of the encapsulated bacteria (Fig. S1B). To evaluate long-term viability of germinated bacteria within the AlgMA matrix, bacterial growth was monitored at 24, 48, 72, and 96 h post-germination (Fig. 2E–H). Given the natural heterogeneity in *B. subtilis* aggregate or microcolony size during bacterial growth, we subdivided aggregates into quartiles based on size distribution. The relative frequency of each quartile at each point was calculated to provide a more nuanced view of population dynamics (Fig. 2E–I-J). Our results indicated that AlgMA was capable of supporting bacterial viability and growth for at least 96 h (Fig. 2K). Notably colony size distribution split bimodally at 24 h, with the highest relative frequencies corresponding to the smallest and largest aggregate sizes. Over time, this distribution became more uniform (Fig. 2K). This behavior is consistent with observations that hydrogel matrix properties directly influence encapsulated microbial colony size and growth dynamics in confined systems [16,28]. The bimodal distribution at 24 h, with the highest frequencies at the smallest and largest aggregate sizes, likely reflects asynchronous spore germination within the AlgMA core, where some spores germinate earlier and rapidly expand while others remain in earlier stages of outgrowth. Over time, this distribution becomes more uniform as smaller colonies continue to grow while larger colonies encounter increasing mechanical confinement from the surrounding matrix, which can restrict further expansion and nutrient diffusion [29,30].

### 2.2 Construction of core-shell structure for metabolite exchange and bacterial entrapment

To construct a core–shell structure capable of physically retaining bacteria while permitting the diffusion of nutrients and bioactive compounds or metabolites, we employed PEGDA (Mn = 250 Da) as the primary structural component of the shell. PEGDA 250 is a widely used material in 3D bioprinting and it forms a dense, non-porous surface when photocrosslinked alone (Fig. S2A). Methanol was incorporated as a porogen to introduce nanoscale porosity within the photocrosslinked PEGDA matrix, inspired by previous work demonstrating the use of porogens to create tunable porosity in photocrosslinkable bioinks [31,32]. We found that increasing the methanol concentration from 0% to 70% (v/v) resulted in progressively larger pore sizes within the PEGDA shell (Fig. S2).

**Fig. 3:**
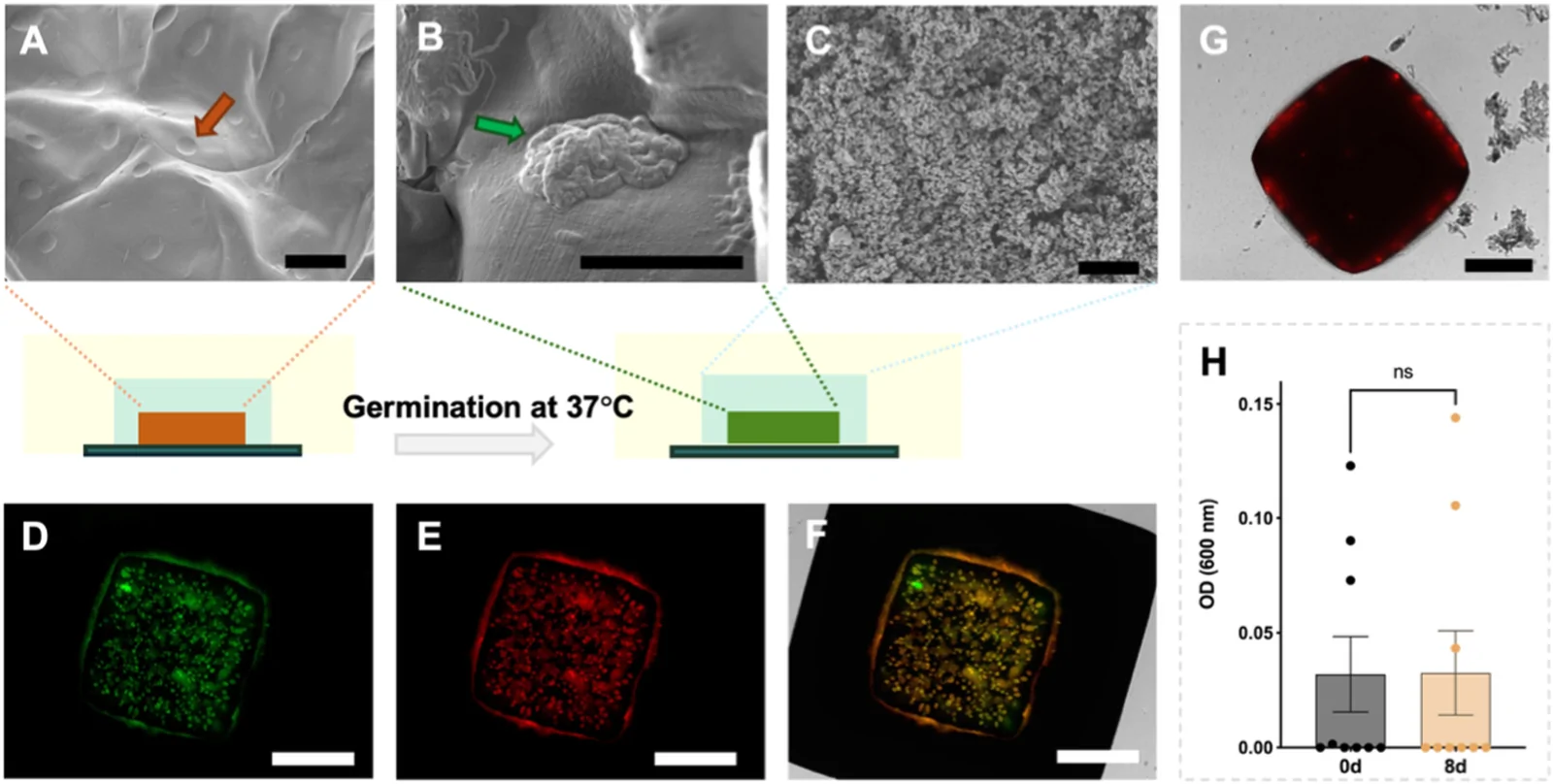
(A-C) SEM images of the spore-encapsulating core before germination (A), after germination (B), and the porous PEGDA-based shell layer (C). The orange arrow indicates an encapsulated spore, while the green arrow highlights germinated bacteria forming a cluster. Scale bar: 10 μm. (D-F) Fluorescence microscopy images of the core–shell structure following germination: GFP signal from live bacteria (D), SYTOX™ Orange staining of non-viable cells after fixation (E), and an overlay with the corresponding bright-field image (F). Spore concentration: 10^7^ spores/mL. Scale bar: 500 μm. (G) SYTOX™ Orange fluorescence from a printed slab composed of shell material mixed directly with 10^7^ spores/mL after germination, showing no bacterial growth within the interior. (H) OD_600_ measurements of supernatant collected from core–shell cultures at day 0 and day 8, demonstrating no significant change in absorbance, and confirming bacterial confinement within the construct (*P* > 0.05; mean ± s.e.m, n = 9).

SEM imaging indicated that spores encapsulated within the AlgMA core successfully germinated into vegetative cells and formed aggregates following culture within the core–shell structure when 70% methanol is used as a porogen (Fig. 3A–C). The PEGDA-based shell exhibited the desired nanoscale pores for entrapping bacteria while allowing molecular diffusion (Fig. 3C) [31]. To further assess cell viability and spatial distribution, we measured GFP fluorescence from live bacteria and, after fixation, SYTOX™ Orange fluorescence from non-viable cells. Most bacteria within the core remained viable (Fig. 3D–F). Importantly, these aggregates were spatially confined within the 1 mm-diameter core region, in alignment with the predefined mask design used during printing. In addition, co-localization of GFP and SYTOX™ Orange signals across the porogen concentration series (0% to 70%) revealed that vegetative cells were only present in the 70% methanol condition, and they were successfully retained (Fig. S3). Quantitative analysis of viable cell counts confirmed high germination efficiency (>99.9%) within the structures, with bacterial growth reaching 10^6^-10^7^ CFU/mL after 8 h (Fig. S4). These results confirm that effective bacterial germination and entrapment were achieved only when sufficient porosity was introduced into the PEGDA shell. Our results indicate 70% methanol is an optimal porogen concentration for enabling controlled permeability while maintaining bacterial confinement.

To further validate the bacterial trapping and nutrient flow capabilities of the shell structure, a control experiment was conducted in which spores were directly mixed into PEGDA shell material at the same concentration used in the core–shell constructs and cultured as before. Only spores located on the surface of the material and with direct access to the culture medium successfully germinated (Fig. 3G). No bacterial colonies were observed within the interior of the PEGDA matrix, in contrast to the encapsulated growth observed within AlgMA cores.

To confirm that the PEGDA shell is required for bacterial containment, we compared supernatant OD_600_ following germination between core–shell constructs and shell-free constructs containing only the AlgMA core. Shell-free constructs showed significantly elevated supernatant OD_600_, indicating bacterial escape into the surrounding medium, whereas core–shell constructs remained at near-baseline OD_600_ (Fig. S5), demonstrating that containment is conferred specifically by the PEGDA shell and cannot be achieved with the AlgMA core alone. Additionally, core–shell constructs were cultured for 8 days to assess long-term bacterial containment. Supernatants were collected at day 0 and day 8 for optical density at 600 nm (OD_600_) measurements. OD_600_ values remained at or near zero for the duration of the experiment and there was no significant difference in OD_600_ between start and end of the experiment (Fig. 3H), indicating *B. subtilis* is unable to grow outside of the structure and is thus effectively trapped. Given that *B. subtilis* can rapidly proliferate and form biofilms in the culture medium used should it have escaped [33], our findings confirm that the core–shell architecture effectively retained viable bacteria within the construct over an 8-day period. To directly quantify molecular transport through the shell, we measured release of tartrazine, a small anionic dye, from core–shell constructs. Tartrazine release increased significantly with porogen concentration and was highest in the absence of a shell (Fig. S6), confirming that the nanoporous PEGDA shell permits porosity-dependent diffusion of small, metabolite-sized molecules while, as shown in Fig. 3G and H, retaining vegetative bacteria.

### 2.3 Influence of structural parameters on bacterial aggregate formation

**Fig. 4:**
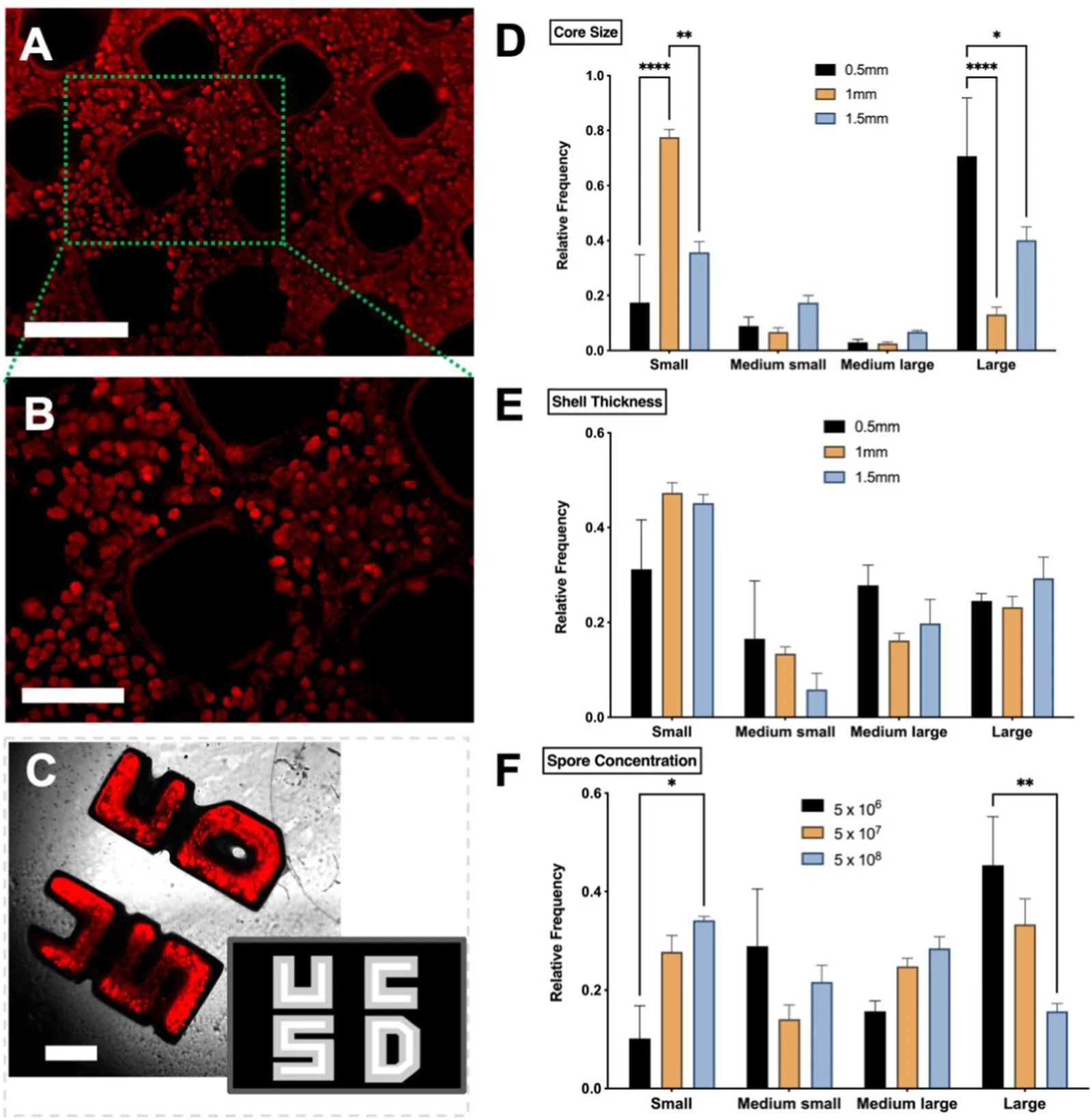
(A) SYTOX™ Orange fluorescence image of a core–shell structure printed using a checkerboard mask pattern, demonstrating uniform spatial distribution of bacterial colonies. Scale bar: 500 μm. (B) Magnified view of the checkerboard pattern showing localized colony distribution. Scale bar: 250 μm. (C) Core–shell structure printed with a custom “UCSD” mask, illustrating high-resolution patterning capability of DLP bioprinting. Scale bar: 500 μm. (D–F) Relative frequency distribution of bacterial colony sizes grouped by quartiles under varying design parameters: (D) different core sizes with constant spore concentration of 10^7^ spores/mL and a fixed shell thickness of 1 mm; (E) different shell thicknesses with a fixed core size of 1.5 mm and spore concentration of 10^7^ spores/mL; and (F) different spore concentrations (5M, 50M, 500M) with fixed core size of 1.5 mm and shell thickness of 1 mm (*P < 0.05, **P < 0.01, ***P < 0.001; mean ± s.e.m, n = 4). Only statistically significant differences are marked; unmarked comparisons were not significant.

Leveraging DLP bioprinting, we fabricated high-resolution, patterned living materials capable of supporting uniform bacterial aggregation across large areas within minutes, achieving substantially higher resolution and throughput compared to millimeter-scale extrusion prints developed to date [34,35]. As shown in Fig. 4A and B, we successfully and rapidly printed “living tiles” using a checkerboard mask pattern, resulting in uniformly distributed bacterial aggregates across a large surface area. These tiles demonstrate the scalability and feasibility of our patterned fabrication method. Additionally, we were able to print high-resolution core–shell structures, wherein the printed core features had an approximate width of 250 μm (Fig. 4C).

To further explore the effects of geometrical and biological parameters on bacterial growth, we systematically investigated the influence of core size, shell thickness, and spore concentration on aggregate formation. In Fig. 4D, core sizes of 0.5, 1, and 1.5 mm were compared under conditions of equivalent spore concentration per unit area and identical shell thickness. Interestingly, opposing trends in aggregate size distribution and core size were observed, which may be attributed to the interplay between total spore number and core surface area. When varying shell thickness while maintaining constant core size and spore concentration, no significant differences were observed across all colony size quartiles, indicating that molecular diffusion through the PEGDA shell remained consistent regardless of tested shell thickness (Fig. 4E). Similarly, core size and shell thickness remained constant while spore concentration was varied (5 × 10^6^, 5 × 10^7^, and 5 × 10^8^ spores/mL) (Fig. 4F). Interestingly, the lowest and highest spore concentrations showed inverse trends in aggregate size distribution with lower spore concentrations favoring the formation of larger aggregates, and higher spore concentrations resulting in more numerous small aggregates. This observation aligns with trends reported by Jeong et al. [36], which suggested quorum-sensing or nutrient competition could influence colony morphology at different initial cell densities.

### 2.4 Resilience of encapsulated *B. subtilis* to common environment stressors

**Fig. 5:**
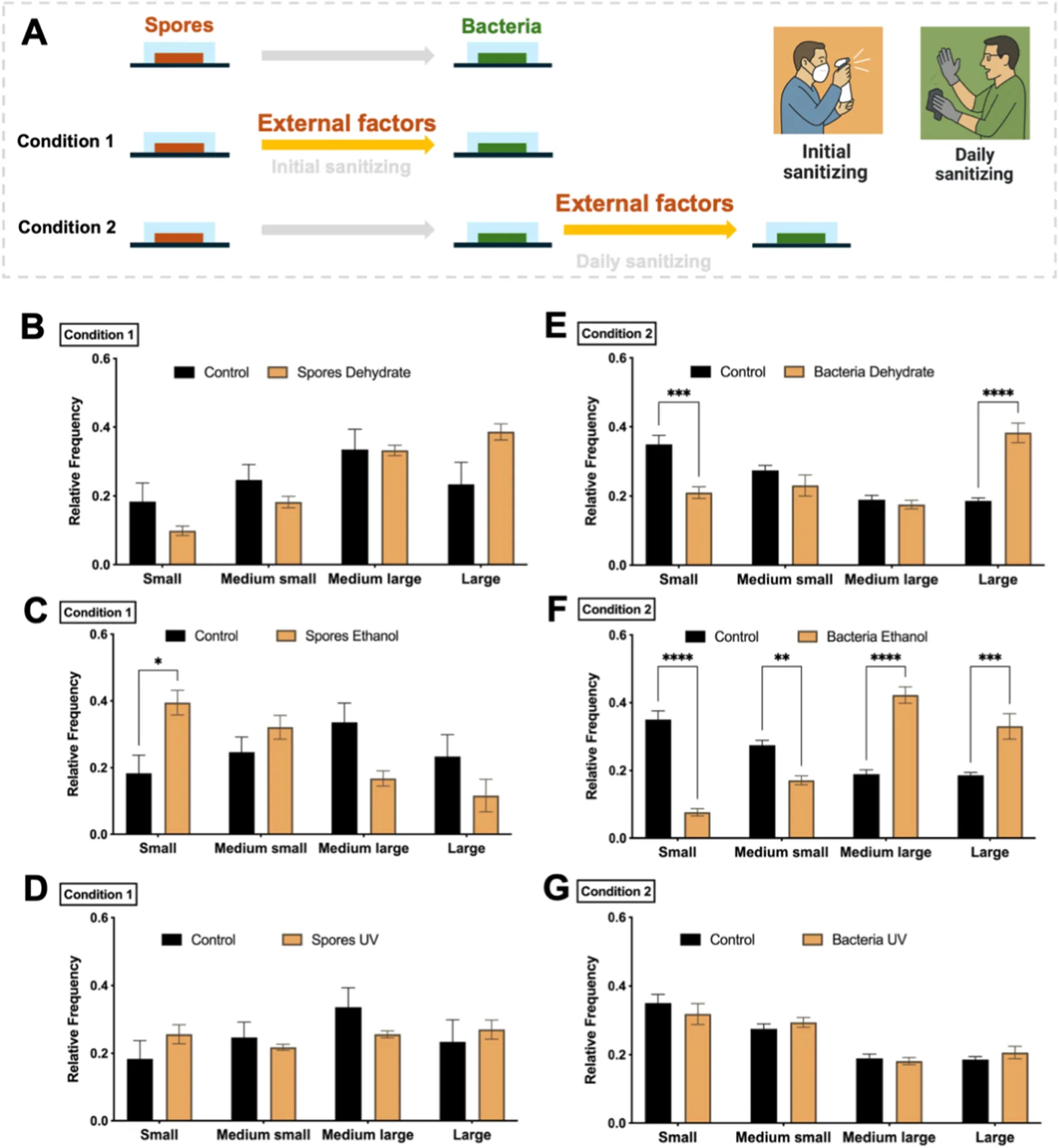
(A) Schematic illustration of experimental design, comparing core–shell structures subjected to external stressors either before (spores) or after (germinated bacteria) germination. (B–D) Colony size distributions of bacteria derived from spores exposed to (B) dehydration, (C) ethanol, or (D) UV-C irradiation. (E–G) Colony size distributions of bacteria exposed to the same stressors after germination and followed by continued incubation: (E) dehydration, (F) ethanol, and (G) UV-C irradiation (*P < 0.05, **P < 0.01; ***P < 0.001; ****P < 0.0001, mean ± s.e.m., n = 4). Only statistically significant differences are marked; unmarked comparisons were not significant.

To evaluate the robustness of the core-shell encapsulation system under realistic application conditions, we systematically investigated the effects of common environmental stressors (UV-C radiation, ethanol exposure, and dehydration) on spore viability and growth. In an initial experiment, we aimed to determine 1) how an initial sanitization process affects spore germination, and 2) how sanitization affects the viability of germinated *B. subtilis* cells, as these structures would likely be exposed to daily sanitization procedures in health-related settings (Fig. 5A). Resulting aggregate size distributions were analyzed for each condition following germination at 37°C for 8h to assess protective efficacy of the core-shell configuration. Control samples not exposed to stressors were also included for each condition. With pre-germination exposure (Condition 1), spores subjected to dehydration or UV-C treatment did not exhibit significant changes in the distribution of germinated bacterial aggregate sizes compared to untreated controls (Fig. 5B and D), suggesting that both the spores and the PEGDA-based shell provide robust protection against desiccation and UV stress. In contrast, ethanol-exposed spores yielded significantly smaller bacterial aggregates post-germination (Fig. 5C). This reduction in aggregate size is consistent with previous findings that ethanol disrupts spore germination [37]. Under post-germination exposure (Condition 2), vegetative cells maintained within the shell structure exhibited stable colony size distributions following UV treatment, indicating that the shell may provide a protective barrier even after germination (Fig. 5G). This protective effect is consistent with the known light-scattering properties of nanoporous polymers, where refractive index mismatch at polymer-pore interfaces attenuates UV transmission through Mie scattering, complementing the intrinsic UV resistance of *B. subtilis* spores [38]. This core–shell system thus preserves viability the of embedded bacteria while still allowing for surface disinfection. When germinated bacteria were exposed to dehydration or ethanol, a marked shift toward larger bacterial clusters compared to control groups was observed (Fig. 5E and F). Dehydration of hydrogel networks has been shown to induce stress-driven reorganization of polymer chains, resulting in matrix densification and altered network architecture, while ethanol exposure can drive sol-to-gel transitions and shrinkage in alginate-based systems, both of which would redistribute the spatial environment available for bacterial aggregation [[39], [40], [41]]. In the core–shell system, the PEGDA shell likely limits the extent of these structural changes, but localized matrix restructuring within the AlgMA core could account for the observed shift in colony size distribution. However, more investigation is necessary to identify the extent hydrogel mechanical properties are altered by each stressor or during bacterial growth. Ultimately, the core-shell system demonstrates distinct protective behaviors depending on whether the biological cargo is in a dormant spore or active vegetative state. In Condition 1, spores remain stable against dehydration and UV-C due to the combined shielding of the PEGDA shell and their own robust structure, though ethanol specifically hinders outgrowth. In Condition 2, the shell continues to protect germinated bacteria from UV, but dehydration and ethanol exposure trigger a shift toward larger bacterial clusters. Our results suggest vegetative cells are likely more sensitive to mechanical alterations in the hydrogel matrix, such as compaction or microcracking, compared to spores.

**Fig. 6:**
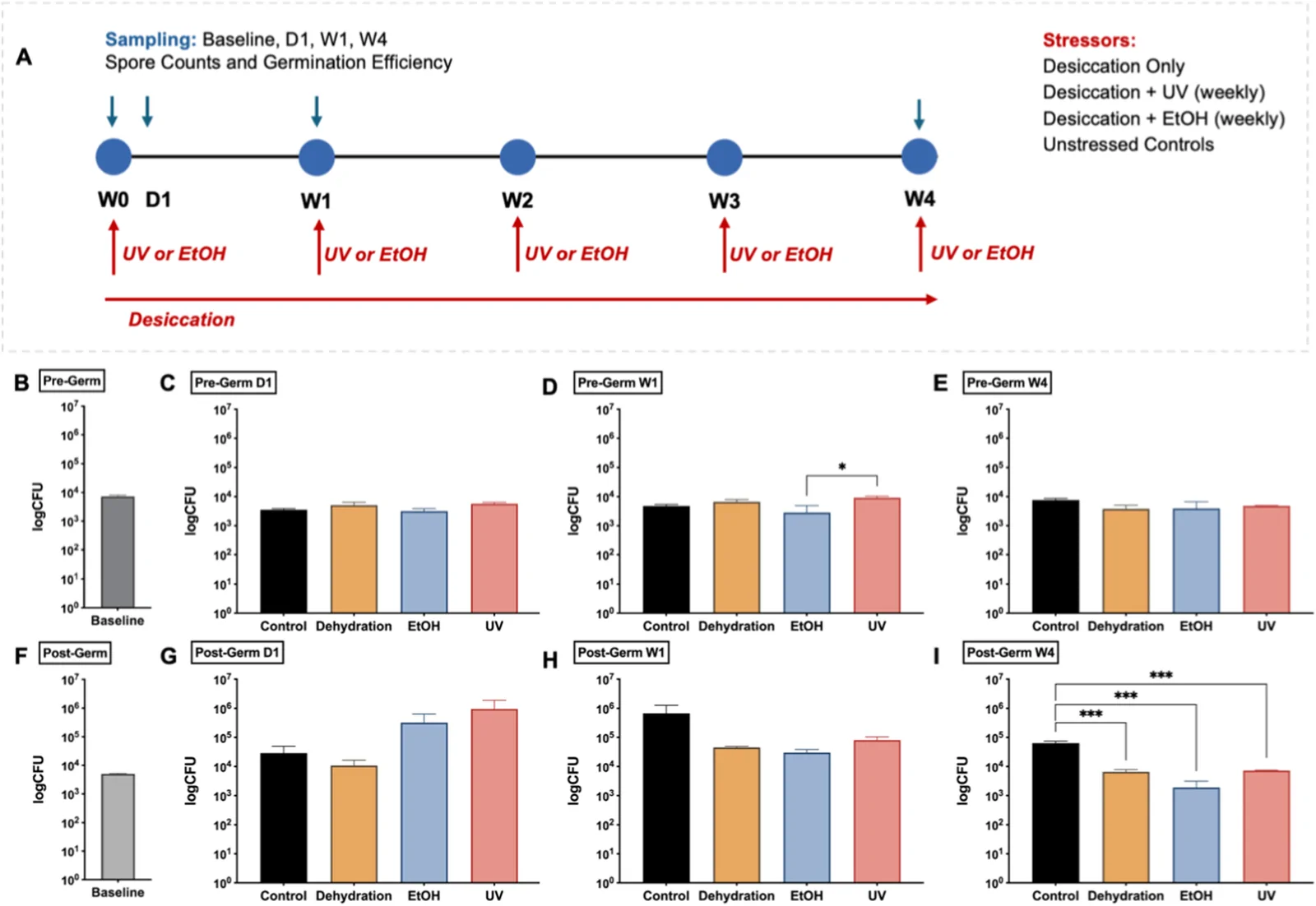
Effects of common environment stressors on *B. subtilis* spore viability and germination with repeated, long-term exposure. (A) Schematic illustration of experimental design. The total number of spores detectable in each structure prior to placing structures in conditions conducive for germination are shown in B-E. The total number of vegetative cells and spores present in each structure following the germination period are shown in F-I. Structures were sampled one day (C, G), one week (D, H), and four weeks (E, I) following initial exposures to the stressors. All data are from one representative experiment (n = 4) and shown as mean CFU/mL±SEM. (*P < 0.05, ***P < 0.001, one-way ANOVA with Tukey's post-test). Only statistically significant differences are marked; unmarked comparisons were not significant.

To further assess resilience under prolonged and repeated stress exposure, we evaluated spore viability after longer, repeated exposure to combinations of the same common environmental stressors. In this subsequent experiment, we assessed total colony forming units (CFU) from spores and vegetative cells at baseline as well as one day, one week, and one month following exposure to UV-C, ethanol, and/or desiccation stress (Fig. 6A). All stressed structures were placed under desiccation stress with ambient temperature and relative humidity for the duration of the experiment, with some structures additionally being subjected to weekly ethanol or UV-C exposure. To determine the number of viable spores remaining in structures at each time point, we enumerated CFU immediately following exposure to each stressor. To determine the extent to which surviving spores could germinate and grow following exposure, a subset of structures was placed in rich media at room temperature and plated for CFU 24 h later.

At baseline (Fig. 6B), there were 7.3 × 10^3^ ± 1.7 × 10^3^ (mean ± standard deviation) spores/mL detectable in structures, and control structures contained between 2.9 × 10^3^ and 1.1 × 10^4^ spores/mL prior to germination throughout the experiment. One day following initial exposures to stress (D1 time point), there were no differences in detectable CFU between any of the stressed conditions relative to controls, both pre-and post-germination (Fig. 6C). After two rounds of ethanol or UV-C exposure (W1 time point), ethanol-exposed structures exhibited a 3.28-fold reduction in detectable spores pre-germination relative to UV-exposed structures (Fig. 6D). This difference suggests ethanol exposure may affect spore viability more quickly than other stressors, although no stressed condition was different from the controls. After five rounds of weekly EtOH or UV-C exposure (W4 time point), there were no significant differences in spore counts between any condition pre-germination (Fig. 6E). In addition, there were no detectable differences between any condition post-germination, suggesting that following initial exposures to ethanol, surviving spores may be able to recover growth (Fig. 6F–H). However, all stressed conditions had significantly fewer detectable CFU following germination relative to the controls, with 9.72, 33.5, and 8.76-fold reductions in CFU/mL for the desiccation only, ethanol, and UV-C-stressed conditions relative to controls, respectively (Fig. 6I). Taken together, these results suggest that the core-shell configuration confers robust short-term protection from multiple stressors, although repeated exposures over time can progressively reduce germination capacity. Specifically, this cumulative sublethal damage appears to manifest primarily during the metabolically demanding germination and subsequent outgrowth processes. Future work should investigate strategies to preserve or restore germination capacity under repeated stress, such as optimizing material formulations for enhanced stress buffering or engineering *B. subtilis* strains with greater stress tolerance during outgrowth. Whether the viable population remaining after repeated stress exposure retains sufficient antimicrobial activity to suppress pathogen growth also remains to be directly evaluated.

### 2.5 Preserved inhibitory activity of encapsulated *B. subtilis* against MRSA

*B. subtilis*-produced antimicrobial compounds have demonstrated activity against *S. aureus* [[42], [43], [44], [45]]. In addition, *B. subtilis* possesses several other competitive mechanisms that could promote greater competitive fitness over pathogens, such as bet-hedging, adopting alternative metabolic states or nutrient acquisition strategies, or simply persisting longer than pathogens [[46], [47], [48]]. To further demonstrate the potential of the proposed core-shell structure for pathogen control, we compared the ability for encapsulated versus free *B. subtilis* spores to germinate and suppress MRSA growth.

**Fig. 7:**
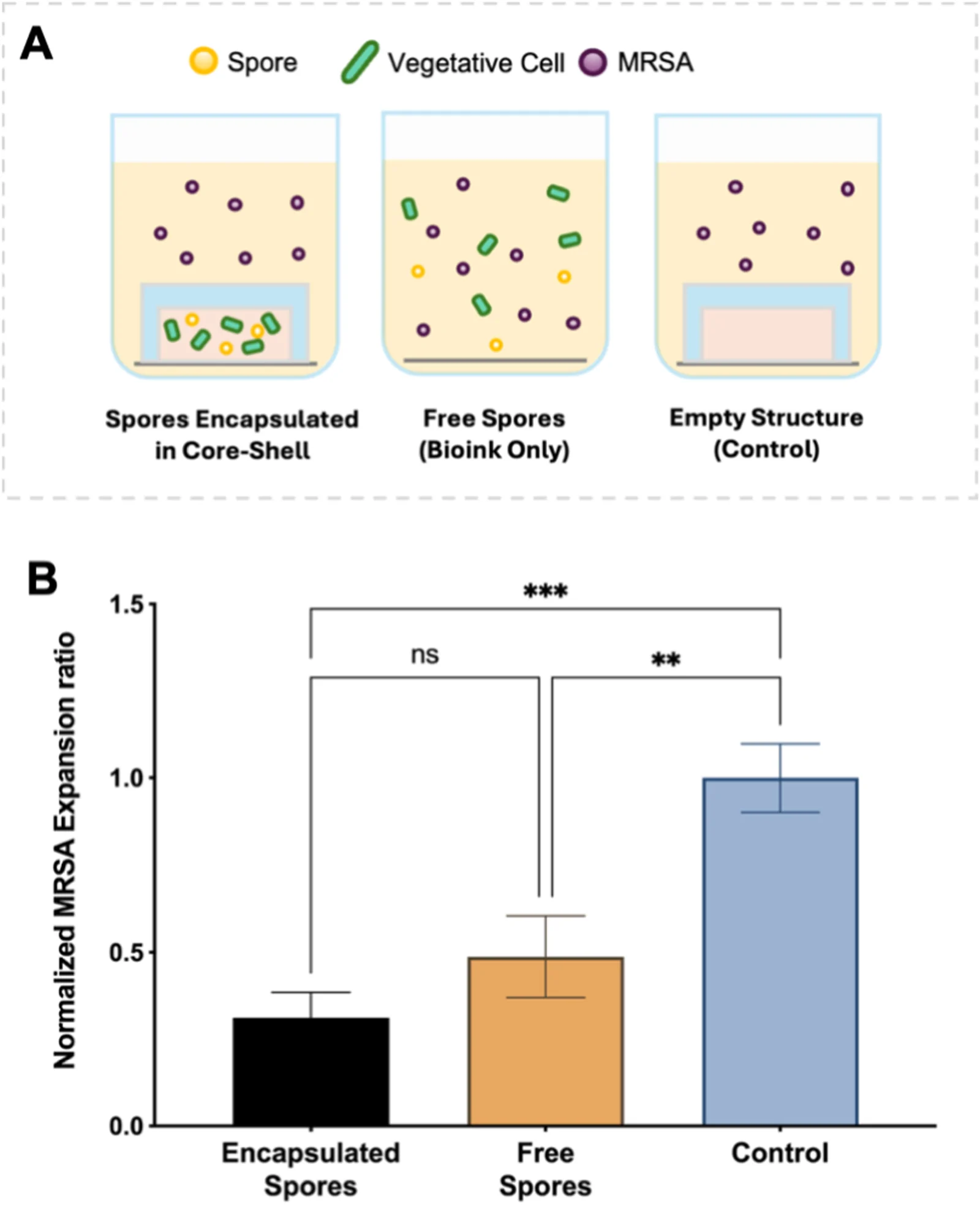
Encapsulated *B. subtilis* suppresses MRSA growth. (A) Schematic of the *anti*-MRSA assay. MRSA is added to media surrounding structures or containing free spores from an equivalent volume of bioink. A control condition containing only core-shell material (no spores) was used to measure uninhibited MRSA growth. *B. subtilis* and MRSA were allowed to grow in co-cultures for 24 h at room temperature before enumerating MRSA colonies. (B) Measured MRSA growth suppression. MRSA expansion ratios were determined by dividing final by initial MRSA CFU/mL for each replicate. Data are normalized relative to average MRSA expansion in conditions with control structures for each experiment. Data are from three independent experiments with n = 4 replicates per experiment. Outliers were removed using an iterative Grubb's test. **P < 0.01, ***P < 0.001, one-way ANOVA with Dunnett's post-test.

MRSA growth was evaluated under three conditions: spores encapsulated within the core-shell structure, free spores able to directly interact with MRSA (bioink only control), and an empty core-shell with no *B. subtilis* to control for uninhibited MRSA growth. MRSA was added to media surrounding structures or containing free spores and allowed to grow for 24 h at room temperature (Fig. 7A). A MRSA growth ratio was used to determine total outgrowth by comparing MRSA CFU/mL at the end of the experiment to CFU/mL in the inoculum. These values are then expressed relative to mean MRSA expansion in the empty core-shell control condition to account for variability across experiments. As shown in Fig. 7B, both the encapsulated spores and free spores demonstrated significant MRSA growth suppression compared to the control group (*P* < 0.001), reducing MRSA expansion by approximately one order of magnitude. Specifically, there was a 3.21-fold reduction in MRSA growth with *B. subtilis* spores encapsulated in the core-shell and a 2.05-fold reduction with free *B*. *subtilis* spores (normalized growth ratios 0.31 ± 0.23 and 0.49 ± 0.41, respectively), relative to the empty core-shell control (normalized growth ratio 1.00 ± 0.34). Notably, no significant difference was observed between the encapsulated and free spore groups, indicating that the core–shell structure does not impede *B. subtilis-*mediated inhibition, facilitating comparable *anti*-MRSA activity to free spores. Our results suggest there is sufficient metabolite exchange through the core-shell to suppress MRSA growth, although the exact mechanism of inhibition remains unresolved. Possible contributing factors include *B. subtilis* antimicrobial metabolite production, quorum sensing interference, nutrient sequestration, or a combination of these and other competitive interactions, and future work will be needed to distinguish among these possibilities. Overall, these results highlight the utility of this material configuration as an encapsulation platform for sustained pathogen suppression, with various potential environmental applications.

## 3 Discussion

Antimicrobial resistance remains a critical global health challenge, and the integration of microbial biocontrol agents for pathogen control in public infrastructure has yet to be fully realized. In this study, we developed a 3D-printed core–shell structure that encapsulates *B. subtilis* spores within biocompatible engineered materials, demonstrating a proof-of-concept approach for long-term, passive biocontrol of antibiotic-resistant pathogens. By leveraging DLP bioprinting, we achieved precise spatial control and scalable fabrication of living architectures with micron-level resolution.

Our material evaluation demonstrates the critical importance of hydrogel selection for bacterial encapsulation systems. The superior performance of natural polymer-based hydrogels over synthetic PEGDA aligns with established principles of biocompatibility in engineered living materials [13,16,27]. The reduced bacterial viability observed in PEGDA is likely attributable to its dense, highly crosslinked network, which restricts nutrient and oxygen diffusion to encapsulated cells, a constraint that has been shown to limit microbial growth in synthetic hydrogel matrices, with cells in denser gels exhibiting reduced proliferation and smaller colony sizes [16,28]. In addition, PEG-based hydrogels are bioinert and lack the cell-interactive cues inherent to natural polymers such as alginate and hyaluronic acid, which provide more permissive microenvironments for microbial survival and growth [28]. The temporal evolution of bacterial aggregate size distributions, from polarized to balanced, reveals complex dynamics between bacterial growth and matrix properties. This behavior suggests that the AlgMA matrix provides an optimal balance between structural support and cellular freedom, enabling controlled bacterial proliferation while maintaining spatial organization. The mechanical feedback mechanisms described by de Souza Heidel et al. [30] provide a theoretical framework for understanding these growth patterns, wherein compressive stress regulation ultimately controls bacterial population dynamics within confined environments.

A key innovation of this work lies in achieving simultaneous biocontainment and functional metabolite release within a single construct—a combination that distinguishes it from prior approaches. Existing spore-based living materials have demonstrated spore inclusion within bulk matrices or bacterial encapsulation in hydrogels, but these systems either lack a physical barrier preventing post-germination bacterial escape or do not demonstrate selective molecular transport through the confining structure [20,21]. Our core–shell architecture addresses both requirements: the AlgMA core supports spore germination and bacterial viability, while the nanoporous PEGDA shell, generated using methanol as a porogen, physically contains vegetative cells while permitting diffusion of nutrients and antimicrobial metabolites. The use of methanol as a porogen to tune this balance between containment and permeability represents an approach that has not been previously explored in living materials [31,32]. While methanol is effective as a proof-of-concept porogen and is used in other biomaterials fabrication contexts such as silk fibroin bioprinting, its toxicity may present challenges for large-scale manufacturing. Future iterations of this platform could explore alternative porogens with lower toxicity profiles to improve scalability and facilitate regulatory compliance [49]. Additionally, a systematic investigation of the porosity-transport-function relationship, including finer resolution mapping of porogen concentration ranges, quantitative pore size characterization, and molecular weight-dependent diffusion profiling, would enable more precise tuning of shell permeability for specific application requirements. Finally, the robust containment demonstrated over 8 days addresses a fundamental concern in living materials by preventing uncontrolled bacterial release while maintaining biological function.

The transition from slow extrusion-based bioprinting to rapid DLP biofabrication represents a paradigm shift in living materials manufacturing [34,35]. Our demonstration of 250 μm resolution features and large-area patterning, plus the potential for roll-to-roll DLP printing addresses scalability concerns that have limited practical implementation of living materials [50]. The systematic study of material parameters revealed previously unreported relationships between structural design and bacterial behavior in confined systems. The concentration-dependent aggregate formation patterns of *B. subtilis* within the core-shell extend quorum sensing principles to engineered encapsulation environments, suggesting that bacterial social behaviors persist even within artificial matrices [36]. The independence of shell thickness on bacterial growth indicates robust mass transport through the porous PEGDA network, supporting the viability of varied architectural designs.

The consistent bacterial viability we observed following exposure to different stressors highlights the robustness of the encapsulation system, and of *B. subtilis* spores, to several relevant environmental challenges. The encapsulation process effectively protected dormant spores, while the altered aggregate distribution patterns of germinated bacteria could be exploited for responsive material design. Given the shift toward larger bacterial aggregates we observed following dehydration, we hypothesize that complete dehydration induces microcracking within the AlgMA hydrogel matrix and creates voids that facilitate bacterial spreading, as consistent with prior findings by Gombotz et al. on hydrogel fracture-mediated colony growth [41]. The ethanol-induced reduction in spore germination we observed is also consistent with known ethanol-mediated disruptions of dipicolinic acid release and cortex degradation [37], while increased bacterial clustering observed with post-germination ethanol exposure suggests potential changes in matrix properties [51,52]. Specifically, the hydrophilic nature of AlgMA makes it susceptible to volume changes when exposed to dehydrating solvents like ethanol, potentially creating a more compact network structure. Finally, as encapsulated *Bacillus* spores germinate and proliferate within the modified core environment, the formation of bacterial biofilms may further influence the local microstructure through physical occupation of pore spaces and secretion of extracellular polymeric substances that can alter the hydrogel matrix properties [51,52]. However, these mechanisms remain speculative and warrant further investigation. Nonetheless, our findings have important implications for the feasibility of deploying this technology in the real world, where materials must withstand diverse environmental conditions while maintaining functionality. Notably, the constructs remained structurally intact throughout the 4-week stress exposure period, and the densely crosslinked PEGDA shell is expected to resist degradation under ambient conditions, although the full operational lifespan and consequences of eventual matrix degradation for containment integrity remain to be characterized.

The maintained antipathogenic activity of *B. subtilis* within the core-shell provides initial evidence supporting the feasibility of this encapsulation approach for pathogen biocontrol applications, though the specific mechanism underlying MRSA suppression has yet to be determined. Our results demonstrate that spatial confinement does not compromise metabolite or bioactive compound exchange, which is an exciting indication of the feasibility of contained yet functional probiotic systems. The reductions in MRSA growth we observed, which are comparable to previous reports of 50-89% MRSA reduction with direct applications of *Bacillus* spores [10], further suggests that encapsulated systems could approach the performance of current probiotic cleaning formulations while providing additional safety benefits via containment. While our work provides an important proof-of-concept for pathogen biocontrol, we acknowledge that future studies are needed to identify the competitive mechanisms contributing to the MRSA growth suppression we observed. Dedicated experiments such as conditioned media assays, metabolite and/or transcript profiling, and work with antimicrobial-deficient strains of *B. subtilis* would be necessary to determine the specific inhibitory mechanisms at play. Nonetheless, the observed MRSA suppression in our study is consistent with our prediction that functional metabolite exchange occurs through the porous shell, supporting the design principle of selective permeability.

While *Bacillus* spores have been used in previous studies, few have achieved spatially confined encapsulation within a protective yet permeable matrix. This core–shell configuration adds a critical biosafety barrier, limiting unintended bacterial release while enabling beneficial functionality post-fabrication. The dormant and highly resilient nature of spores further supports decentralized manufacturing and long-term storage. This design presents opportunities for sustained antimicrobial activity across a range of potential applications while expanding the functional repertoire of living materials. In future work, *B. subtilis* could be genetically engineered to further promote its persistence, nutrient acquisition, and antipathogenic activity to reduce pathogen transmission in environmental settings such as healthcare facilities, food processing, and public infrastructure. Additionally, *B. subtilis* could be engineered to respond to specific environmental cues, such as the presence of pollutants or of specific signaling molecules, to enable new functionalities in biosensing, biodegradation, or therapeutic delivery.

Importantly, we note that the present study is intended as a conceptual demonstration under controlled laboratory conditions. Across environmental stressor and MRSA inhibition experiments, *B. subtilis* spore germination, growth, and inhibitory activity were achieved within 8 h at 37°C and within 24 h at ambient temperature, all under nutrient-rich conditions. Moreover, encapsulated spores survived up to 4 weeks of desiccation while retaining germination capacity upon rehydration. These results suggest the platform is most compatible with environments where intermittent moisture or nutrient availability can support germination, such as humid indoor surfaces or biomedical packaging in contact with aqueous environments. However, further work is needed to evaluate germination, growth, and pathogen inhibition under application-specific conditions, including with nutrient limitation and under ambient humidity. In particular, future studies should benchmark biocontrol efficacy against established antimicrobial strategies, assess long-term structural and functional stability under realistic deployment scenarios, and evaluate containment robustness under mechanical and chemical challenges representative of target environments. Additionally, evaluating *B. subtilis*-mediated pathogen inhibition with varied pathogen inoculum levels, repeated challenge conditions, and extended time points, as well as against a broader panel of drug-resistant pathogens beyond MRSA, will be important for establishing the generalizability and durability of the biocontrol approach. The influence of local transport conditions on antimicrobial efficacy or metabolite accumulation also warrants investigation, as dynamic flow environments may continuously remove secreted metabolites from the material surface, potentially reducing local inhibitory concentrations compared to the static co-culture conditions used in the present study. Ultimately, in harnessing the inherent robustness of *B. subtilis* spores and the precision of DLP bioprinting, this work lays the foundation for scalable, programmable, and resilient living materials that address public health challenges across environmental and biomedical contexts.

## 4 Materials and methods

### 4.1 Chemicals

PEGDA (Mn = 700 Da and Mn = 250 Da), methacrylic anhydride (MA), tartrazine, sodium alginate, N-hydroxysuccinimide (NHS), glycidyl methacrylate, triethylamine (TEA), and isopropyl alcohol (IPA) were obtained from Sigma–Aldrich (St. Louis, MO, USA). Sodium chloride (NaCl), isopropyl-β-D-thiogalactopyranoside (IPTG), methanol, paraformaldehyde, and SYTOX™ Orange nucleic acid stain were purchased from Thermo Fisher Scientific (Waltham, MA, USA). Lithium phenyl-2,4,6-trimethylbenzoylphosphinate (LAP), 2-morpholinoethanesulfonic acid (MES), and 1-ethyl-3-(3-dimethylaminopropyl)carbodiimide hydrochloride (EDC) were obtained from Tokyo Chemical Industry (Tokyo, Japan). Hyaluronic acid (HA; molecular weight range: 10 kDa to ∼1.0 MDa) was sourced from Bloomage Biotechnology (Jinan, China). 2-Aminoethyl methacrylate hydrochloride (AEMA) was purchased from Acros Organics (Geel, Belgium). Dulbecco's phosphate-buffered saline (DPBS) was obtained from Gibco (Thermo Fisher Scientific).

### 4.2 Hydrogel synthesis

AlgMA hydrogel was synthesized following previously published protocol [53]. Briefly, a 1% (w/v) sodium alginate solution was prepared in a 50 mM MES buffer containing 0.5 M NaCl, adjusted to pH 6.5. The carboxyl groups on the alginate backbone were activated by the addition of NHS and EDC for 5 min. Subsequently, AEMA was introduced and allowed to react for 24 h. A 1:2:1 M ratio of NHS:EDC:AEMA was used, with AEMA added at a concentration of 2.3 mmol per gram of sodium alginate. The reaction was quenched by precipitation in excess acetone, and the resulting product was dissolved in Milli-Q water, followed by dialysis against Milli-Q water using 12–14 kDa molecular weight cutoff tubing for 3 days. The dialyzed product was then lyophilized and stored. All synthesis and purification steps were conducted at room temperature.

Hyaluronic acid glycidyl methacrylate (HAGM) was synthesized via a modified procedure based on previously reported methods [54]. In brief, 1 g of HA was dissolved in 100 mL of a 1:1 (v/v) acetone:water mixture and stirred overnight. A volume of 1.8 mL TEA was added dropwise and allowed to mix thoroughly, followed by the dropwise addition of 1.8 mL glycidyl methacrylate. The reaction was allowed to proceed overnight with continuous stirring. The reaction mixture was then transferred to 3500 Da molecular weight cutoff dialysis tubing and dialyzed against deionized water for 48 h, with water changes three times per day. The final product was frozen and lyophilized for storage.

### 4.3 Bacterial strains and growth conditions

*B. subtilis* strain TH035, a soil isolate, was selected as the wildtype strain and obtained from the *Bacillus* Genetic Stock Center (BGSC). An inducible GFP expression plasmid was introduced into this strain background to generate *B. subtilis* TH035 (pSC01-GFP), as detailed below. Methicillin resistant *Staphylococcus aureus* subsp. *aureus* Rosenbach TCH1516, a clinical isolate, was obtained from the American Type Culture Collection (ATCC; BAA-1717). Both MRSA and *B. subtilis* were routinely cultured in tryptic soy broth (TSB; 1.7% wt/vol pancreatic casein peptone, 0.3% wt/vol soya peptone, 0.5% wt/vol sodium chloride, 0.25% wt/vol dipotassium hydrogen phosphate, 0.25% wt/vol glucose), with CFU enumerated on tryptic soy agar (TSA). *B. subtilis* and MRSA cultures were routinely incubated at 30 and 37°C, respectively, and MRSA inhibition experiments were conducted at room temperature, as described below.

### 4.4 *B. subtilis* strain and plasmid construction

To improve microscopic visualization of *B. subtilis* TH035, it was transformed with a plasmid which enables high levels of GFP expression, pSC01 (Table 1). The pSC01 plasmid was constructed by modifying the shuttle plasmid pLIKE-sipW-tasAss(30)-silk (10Ala)1 [55]. Regulatory elements were introduced, and nonsense sequences within the template plasmid were removed to improve plasmid stability. Initially, the template plasmid was sequenced using Nanopore sequencing (Plasmidsaurus Inc.) to confirm its whole sequence. The plasmid was then digested with *Hin*dIII and *Sap*I restriction enzymes to excise nonsense regions. The resulting linearized plasmid was ligated to eliminate redundant sequences. To generate the new plasmid backbone, the modified construct was PCR-amplified using primers pSC-BB-F and pSC-BB-R. In parallel, a promoter-operator fragment was amplified using primers O1-PgroES-CosE-F and O1-PgroES-CosE-R, and a GFP coding sequence was amplified from a pBAD-GFP plasmid using primers GFP-F and GFP-R. These three fragments—the backbone, regulatory element, and GFP insert—were assembled via Gibson assembly. The assembled plasmid was transformed into 10-beta Competent *E. coli* cells (NEB), and colonies were screened for correct constructs. Verified clones were cultured, and plasmids were extracted for subsequent experiments.

**Table 1:** List of oligonucleotides used in plasmid construction.

pSC-BB-R	CATgaattcaattgttatccgctcacaattccatcctccaaagttggagagtgag
pSC-BB-F	GACGTCTTTGAGAATAGGGAGTTGAGCGTATTTGCTTATACTC CTTATTTTCTTTTTTTTccggtggaaacgaggtc
O1-PgroES-CosE-F	tgagcggataacaattgaattcATGGTATGTACTCCTTTGTTAAGTGGG
O1-PgroES-CosE-R	TTGCTAACCATctagAATCCTCCTTAACTAATTGGTGTTGG
GFP-F	TAAGGAGGATTctagATGGTTAGCAAAGGTGAAGAACTGTTTAC
GFP-R	ATTCTCAAAGACGTCTTAATGGTGATGATGATGGTGGCTGC

This plasmid was then transformed into *B. subtilis* strain TH035 using a natural competency protocol. In short, an overnight culture of *B. subtilis* in Lysogeny broth (LB) was diluted 1:10 in MNGE media (prepared as described by Tartık) to induce competence. This {Citation}culture was grown at 37 until an OD600 of 0.5-0.7 was reached as measured via Genesys 10S UV-Vis Spectrophotometer (Thermo Fisher Scientific). 400 L of culture was taken, 1-2 μg of purified pSC01 plasmid was added, and that culture was grown for 1 h. At this point, 100 L of expression mix was added (prepared as described by Tartık), the culture was grown for an additional hour, then plated on LB agar plates with 5 g/mL chloramphenicol as a selection marker [56]. Colonies were selected based on visible GFP expression relative to a wildtype control and validated using colony PCR to amplify the inserted region.

### 4.5 *B. subtilis* spore purification

*B. subtilis* TH035 (pSC01-GFP) spores were isolated from cultures grown in Difco Sporulation medium (DSM) for 72 h at 37°C. DSM was prepared by combining Difco nutrient broth, consisting of 0.3% wt/vol beef extract and 0.5% wt/vol peptone, with 0.1% wt/vol KCl and 0.012% wt/vol MgSO_4_⋅7H_2_O; the entire mixture was adjusted to pH 7.6 prior to autoclaving. Post-autoclave, Ca(NO_3_)_2_, MnCl_2_, and FeSO_4_ were added to achieve final concentrations of 1 mM, 0.01 mM, and 1 μM, respectively. Following growth in this medium for 72 h, bacterial mass was collected via centrifugation and washed in sterile water over the course of two days. Renografin purification was then performed to separate the spores from vegetative cells. Briefly, *B. subtilis* suspensions were added to a two-phase extraction system consisting of 34% v/v 3M potassium phosphate buffer and 11.18% v/v 50% polyethylene glycol (PEG) 4000, as described [57]. After vigorously mixing the solution for 15 min and centrifuging, the top phase was collected. The extracted top phase was then diluted with sterile water and pelleted; this process was repeated five more times to remove any residual PEG 4000. Isolated spores were further purified via centrifugation through a 20% to 50% HistoDenz gradient, as described [58], to select only dormant, phase-bright spores. Final spore concentrations were determined by plating dilutions on TSA [58].

### 4.6 3D printing of spores encapsulated core-shell structure

A bioink consisting of 2% (w/v) AlgMA, 0.25% (w/v) LAP, and a defined concentration of *B. subtilis* spores suspended in DPBS was used throughout the study. Unless otherwise specified, the spore concentration in the bioink was maintained at 10^7^ spores/mL. Bioprinting was conducted using a custom-built DLP bioprinter equipped with a 385 nm light source. The bioink reservoir was assembled using glass slides and polydimethylsiloxane (PDMS) sheets, while methacrylate-coated coverslips served as the substrate for printing. The core structure was printed using a light intensity of 5.57 mW/cm^2^ with a 10-s exposure, resulting in a 125 μm thick construct. The shell structure was printed under the light conditions of 2.28 mW/cm^2^ for 10 s to achieve a thickness of 250 μm. Following core structure fabrication, the printed samples were immediately rinsed three times with DPBS. The shell material was then added to the reservoir and photopolymerized. Upon completion, the printed constructs were rinsed three times with IPA, followed by three additional rinses with PBS to remove residual unpolymerized materials.

### 4.7 Spore quantification and germination in printed structures

Following printing, we confirmed the actual number of spores within each structure prior to inducing germination. Briefly, core-shell structures were submerged in DPBS and a scalpel was used to expose the spore-embedded core. A bioink only condition was also used as a control to assess the extent to which all spores could be recovered from the structures. A solution of alginate lyase in DPBS was added to all samples achieve a final concentration of 1 mg/mL, and the entire mixture incubated for 30 min at 150 RPM to release all spores from the core-shell or bioink. Following release, spores/mL were enumerated on TSA. We then wanted to determine the efficiency of spore germination and extent of outgrowth within structures or from bioink. To induce germination, sample structures and/or bioink only controls were cultured in a medium composed of 90% tryptic soy broth (TSB) and 10% DPBS. To enumerate spores and vegetative cells in structures or in bioink, cultures were incubated at 37°C and 150 RPM for 8 h prior to plating CFU as above. To obtain counts of spores only for determining germination efficiency, a subsample from each structure was heat-shocked at 80°C for 10 min to lyse any vegetative cells. For microscopy, 90% TSB media was supplemented with 1 mM isopropyl β-D-1-thiogalactopyranoside (IPTG) in DPBS to induce GFP expression. Cultures were then incubated at 37°C with agitation at 250 revolutions per minute (rpm) for 8 h.

### 4.8 Mechanical characterization

Mechanical properties of the core hydrogel material were assessed using a universal mechanical testing machine (MicroTester G2, CellScale, Canada). Cylindrical samples with a diameter and height of 500 μm were prepared and placed on the testing platform. A compression test was performed at a constant displacement rate of 0.08 mm/s, and force-displacement data were recorded to evaluate the compressive response of the printed hydrogel, then Young's modulus was calculated using an in-house MATLAB code.

### 4.9 Tartrazine diffusion assay

To directly assess small-molecule transport through the PEGDA shell, tartrazine (MW 534 Da) was used as a model small-molecule diffusant. Three construct types were compared: core–shell constructs with a 0% (v/v) methanol shell, core–shell constructs with a 70% (v/v) methanol shell, and a no-shell control consisting of the AlgMA core alone. Tartrazine was incorporated into the AlgMA core bioink at a final concentration of 0.1 mg/mL prior to printing, and constructs were fabricated as described above. Following fabrication, constructs were rinsed 3 times with IPA and DPBS to remove surface-associated dye and then transferred individually into 1 mL of DPBS and incubated with gentle shaking. At 8h, an aliquot of the surrounding solution was collected, and tartrazine concentration was determined from absorbance at 425 nm using a microplate reader curve. Relative tartrazine release (%) was calculated as the supernatant absorbance of each sample divided by that of the blank, expressed as a percentage.

### 4.10 Microscopy and image analysis

Bright-field and fluorescence imaging were performed using an inverted fluorescence microscope (Leica DMI 6000B) and a laser scanning confocal microscope (Leica SP8). For microstructural characterization, hydrogel samples were freeze-dried and analyzed using a field-emission scanning electron microscope (SEM, Zeiss Sigma 500). Following germination, samples were rinsed three times with phosphate-buffered saline (DPBS), and green fluorescent protein (GFP) fluorescence was recorded to identify viable vegetative cells originating from germinated spores. Subsequently, the samples were fixed using 4% (w/v) paraformaldehyde in PBS. For total bacterial labeling, including both live bacteria and ungerminated spores, 500 nM SYTOX™ Orange was added to label DNA [59,60]. SYTOX™ Orange fluorescence was captured using excitation at 540–552 nm and emission at 580–620 nm, while GFP fluorescence was detected using 460–500 nm excitation and 512–542 nm emission filters. Together, this dual-labeling approach enables the differentiation between live vegetative *B. subtilis* (GFP-positive) and the total bacterial population (SYTOX™ Orange-positive), with dead bacteria after germination presumed to be SYTOX™ Orange-positive but GFP-negative.

Image analysis was conducted using ImageJ software. For Fig. 2D, fluorescence intensity was quantified using the straight line and plot profile tools, and intensity density values were normalized to the PEGDA-only group, expressed as “relative intensity density”. For bacterial aggregate distribution analysis, as shown in Fig. 2E–I-J, images were converted to binary and assessed using the built-in watershed analysis tool, then measured with the built-in particle analyzer tool to quantify the aggregate sizes, and the data were grouped into quartiles: Small, Medium small, Medium large, and Large. The frequency of each subgroup under specific conditions was calculated and reported as “relative frequency” to illustrate differences in spatial distribution and colony growth dynamics within the hydrogel core.

### 4.11 Environmental stressor exposure experiments

The exposure experiments were designed to simulate common environmental stressors relevant to potential deployment contexts. To determine how initial sanitization can affect spore germination as well as germinated cell viability, three experimental groups were established: (1) the control group, in which spore-encapsulated core–shell structures were germinated at 37°C for 8h as described above; (2) the spore exposure group, where encapsulated spores were subjected to environmental stressors prior to germination; and (3) the bacteria exposure group, in which the core shell structures were placed in TSB medium and germinated overnight, followed by gentle washing with DPBS three times and subsequent exposure to the same environmental conditions. Environmental stressors included UV-C light, 100% ethanol, and dehydration. For UV-C exposure, structures were irradiated for 1 h using UV-C lamps emitting at 254 nm. Ethanol treatment was performed by immersing the structures in 100% ethanol for 1 h. For the dehydration/rehydration experiment, structures were dried overnight in a 37°C oven and then rehydrated in PBS for 2 h prior to subsequent analysis. All structures were assessed for bacterial aggregate distribution using the microscopy and image analysis procedures described above.

For the long-term stressor exposure experiment, spore-encapsulated core–shell structures were subjected to extended environmental stress conditions over a 4-week period with sampling at day 1 (D1), week 1 (W1), and week 4 (W4). Four treatment conditions were established: (1) control structures maintained in sterile water throughout the experiment with water refreshed weekly, (2) dehydrated structures that were air-dried and stored under ambient conditions, (3) dehydrated structures that additionally underwent weekly ethanol treatment as above, and (4) dehydrated structures that additionally received weekly UV-C irradiation as above.

At baseline and immediately following each post-exposure sampling time point (D1, W1, W4), four replicate structures from each condition were directly opened, incubated with alginate lyase to release spores from bioink as described above, and plated on TSA for CFU enumeration to determine the total number of spores present in each structure. These data are referred to as pre-germination spores/mL. We additionally determined the capacity for the spores in each structure to germinate and grow under conditions with rich media, but ambient temperature (24°C), which was used to more closely mimic potential germination conditions under ambient deployment scenarios. Briefly, a separate set of four replicate structures from each condition were placed in 1 mL of 90% TSB and left shaking at 150 RPM for 24 h at 24°C. Following incubation, CFU from vegetative cells and spores were enumerated as described above, and the resulting data are referred to as post-germination CFU/mL. This dual approach enabled differentiation between spore survival and functional germination capacity under long-term environmental stress conditions.

### 4.12 MRSA inhibition assays

We evaluated the capacity for *B. subtilis* spores embedded in structures to inhibit the growth of MRSA subsp. *aureus* TCH1516 (ATCC BAA-1717). Structures containing approximately 5 × 10^5^ spores or control structures without spores were submerged in a solution of 90% TSB media and 10% DPBS containing approximately 10^2^ CFU/mL MRSA. Free spore competition control conditions with the same concentration of MRSA and 1 μl bioink (containing the same number of spores as in core-shell structures) were also included. As a control for uninhibited MRSA growth, a condition with only shell material (no spores) was included. All samples were cultured at room temperature (24°C) for 24 h with gentle shaking (120 RPM). This temperature was used to more closely mimic ambient environmental conditions. At the end of the experiment, all samples were treated with 180 Kunitz DNaseI (Zymo DNaseI Set) and allowed to incubate at 37°C for 1 h with shaking (150 RPM) to disperse any MRSA biofilms and facilitate accurate CFU quantification. MRSA CFU/mL were then quantified from media surrounding structures or bioink by plating dilutions on TSA with 4 mg/mL methicillin.

Statistics and reproducibility.

All statistical analyses were performed using Prism 9 (GraphPad Software, La Jolla, CA, USA). The normality of the data was assessed using the Shapiro–Wilk test to confirm the appropriateness of parametric tests. For multiple comparisons, a one-way analysis of variance (ANOVA) followed by post hoc Sidak multiple comparisons tests was conducted. A significance threshold (α) of 0.05 was applied for all statistical tests. Data are presented as mean ± standard deviation unless otherwise specified.

## CRediT authorship contribution statement

**Lin Huang:** Writing – review & editing, Writing – original draft, Visualization, Validation, Methodology, Investigation, Formal analysis, Data curation, Conceptualization. **Kathleen L. Furtado:** Writing – review & editing, Visualization, Validation, Methodology, Investigation, Data curation. **William Brakewood:** Resources. **Maxwell Neal:** Resources. **Yazhi Sun:** Visualization. **Jasmine Le:** Investigation. **Qi Xie:** Resources. **Jacob Hizon:** Investigation. **Shivam Singhal:** Investigation. **Mariana Salas Garcia:** Investigation. **Joshua Tran:** Investigation. **Karsten Zengler:** Investigation. **Michael Betenbaugh:** Investigation. **Jack A. Gilber:** Writing – review & editing, Supervision, Project administration, Funding acquisition. **Shaochen Chen:** Writing – review & editing, Supervision, Project administration, Funding acquisition.

## Ethics approval and consent to participate

This article does not contain any original studies with human participants or animals performed by any of the authors. Therefore, ethical approval and consent are not applicable.

## Data availability

All data needed to evaluate the conclusions in the paper are present in the paper and/or the Supplementary Materials.

## Declaration of competing interest

The authors declare that they have no competing interests.

## Footnotes

Appendix A Supplementary data to this article can be found online at https://doi.org/10.1016/j.bioactmat.2026.08.025.
