# Supplementary material for: Bioprinted core-shell living material platform for spatially controlled encapsulation of Bacillus subtilis and sustained metabolite exchange
Source: Bioact Mater. 2026 Aug 31;68:82–95. doi: 10.1016/j.bioactmat.2026.08.025 (PMC13562397; doi:10.1016/j.bioactmat.2026.08.025)
Supplement: Supplementary file 1 [file mmc1.docx]

**Supporting Information for**

**Bioprinted Core-Shell Living Material Platform for Spatially Controlled Encapsulation of *Bacillus subtilis* and Sustained Metabolite Exchange**

*Lin Huang ^1†^, Kathleen L. Furtado^3,4†^, William Brakewood ^7^, Maxwell Neal ^3^, Yazhi Sun ^1^, Jasmine Le ^2^, Qi Xie^8^, Jacob Hizon^4^, Shivam Singhal^4^, Mariana Salas Garcia^4^, Joshua Tran^3,4^, Karsten Zengler ^2, 3, 5, 6^, Michael Betenbaugh ^7^, Jack A. Gilbert^3, 4, 5*^, Shaochen Chen ^1,2*^*

L. Huang, YZ. Sun and S. Chen

Department of Chemical and Nano Engineering, University of California San Diego, La Jolla, USA

Email: [shc064@ucsd.edu](mailto:shc064@ucsd.edu)

J. Le, K. Zengler and S. Chen

Department of Bioengineering, University of California San Diego, La Jolla, USA

Email: [shc064@ucsd.edu](mailto:shc064@ucsd.edu)

K. L. Furtado, M. Neal, J. Tran, K. Zengler and J. A. Gilbert

Department of Pediatrics, University of California San Diego, La Jolla, CA, USA

Email: [gilbertjack@gmail.com](mailto:jagilbert@health.ucsd.edu)

K. L. Furtado, J. Hizon, S. Singhal, M. S. Garcia, J. Tran and J. A. Gilbert

Scripps Institution of Oceanography, University of California San Diego, La Jolla, CA, USA

Email: [gilbertjack@gmail.com](mailto:jagilbert@health.ucsd.edu)

K. Zengler and J. A. Gilbert

Center for Microbiome Innovation, University of California San Diego, La Jolla, CA, USA

Email: [gilbertjack@gmail.com](mailto:jagilbert@health.ucsd.edu)

K. Zengler

Program in Materials Science and Engineering, University of California San Diego, La Jolla, CA, USA

W. Brakewood and M. Betenbaugh

Department of Chemical and Biomolecular Engineering, Johns Hopkins University, Baltimore, MD, USA

Q. Xie

Department of Chemistry, Johns Hopkins University, Baltimore, MD, USA

*Correspondence: Prof. Shaochen Chen; email: [shc064@ucsd.edu](mailto:shc064@ucsd.edu); Prof. Jack Gilbert, email: [gilbertjack@gmail.com](mailto:jagilbert@health.ucsd.edu)

^†^These authors contributed equally to this work.

**Figure S1:** Altering light intensity during fabrication does not affect the mechanical properties of structures nor bacterial viability. (A) Young’s modulus of AlgMA constructs printed under different light intensities. (B) Relative GFP fluorescence intensity of bacteria encapsulated in the AlgMA core printed under different light intensities.

**Figure S2:** Increasing concentrations of methanol improve the apparent porosity of outer shell material. Scanning electron microscopy (SEM) images of PEGDA 250 morphologies with methanol concentrations of 0% (A), 30% (B), 50% (C), and 70% (D) v/v. Scale bar: 10 μm.

**Figure S3:** *B. subtilis* spores only germinate when 70% methanol is used as a porogen. Overlay images of GFP fluorescence, SYTOX™ Orange staining, and bright-field microscopy of core–shell structures. The shell structures are composed of PEGDA 250 incorporating 0% (a), 30% (b), 50% (c), and 70% (d) methanol (v/v) as a porogen. Scale bar: 500 μm.

**Figure S4:** Viable cell counts of *B. subtilis* spores and vegetative cells in bioink controls versus spores encapsulated structures before and after germination under nutrient-rich conditions for eight hours at 37°C. Data are from one representative experiment.

**Figure S5.** OD₆₀₀ of supernatant collected after germination from core–shell constructs (Shell) versus shell-free AlgMA core-only constructs (No shell) (****P < 0.0001; mean ± s.e.m., n = 9).

**Figure S6.** Relative tartrazine release (%) from core–shell constructs with a 0% porogen shell, a 70% porogen shell, and a no-shell control, used as a small-molecule diffusion probe (P < 0.05, **P < 0.01, ****P < 0.0001; mean ± s.e.m., n = 6).
